# Variation of PEAR1 DNA methylation influences platelet and leukocyte function

**DOI:** 10.1186/s13148-019-0744-8

**Published:** 2019-10-29

**Authors:** Benedetta Izzi, Francesco Gianfagna, Wen-Yi Yang, Katrien Cludts, Amalia De Curtis, Peter Verhamme, Augusto Di Castelnuovo, Chiara Cerletti, Maria Benedetta Donati, Giovanni de Gaetano, Jan A. Staessen, Marc F. Hoylaerts, Licia Iacoviello, on behalf of Moli-family Investigators, Licia Iacoviello, Branislav Vohnout, Marcello Arca, Chiara Cerletti, Maria Benedetta Donati, Giovanni de Gaetano, Roberto Lorenzet, Augusto di Castelnuovo, Simona Costanzo, Francesco Gianfagna, Romina di Giuseppe, Antonella Cutrone, Amalia De Curtis, Sara Magnacca, Benedetta Izzi, Marilena Crescente, Agnieszka Pampuch, Chiara Tamburrelli, Emanuela Napoleone, Filomena Zurlo, Luisa Nanni

**Affiliations:** 1 Department of Epidemiology and Prevention, IRCCS NEUROMED, Via dell’Elettronica, 86077 Pozzilli, IS Italy; 2 Mediterranea Cardiocentro, Naples, Italy; 3 Department of Medicine and Surgery, University of Insubria, Varese, Italy; 4 Studies Coordinating Centre, Research Unit Hypertension and Cardiovascular Epidemiology, Department of Cardiovascular Sciences, University of Leuven, Leuven, Belgium; 5 Department of Cardiovascular Sciences, Center for Molecular and Vascular Biology, University of Leuven, Leuven, Belgium

## Abstract

**Background:**

Platelet-endothelial aggregation receptor 1 (PEAR-1) is a transmembrane receptor involved in platelet activation and megakaryopoiesis whose expression is driven by DNA methylation. *PEAR1* variants were associated with differential platelet response to activation and cardiovascular outcomes. We aimed at investigating the link between *PEAR1* methylation and platelet and leukocyte function markers in a family-based population.

**Results:**

We measured *PEAR1* methylation in 605 Moli-family participants with available blood counts, plasma P-selectin and C-reactive protein, whole blood platelet P-selectin, and platelet-leukocyte mixed conjugate measurements. We performed principal component analysis (PCA) to identify groups of highly correlated CpG sites. We used linear mixed regression models (using age, gender, BMI, smoking, alcohol drinking, being a proband for family recruitment, being a member of myocardial infarction (MI) family as fixed effects, and family as a random effect) to evaluate associations between *PEAR1* methylation and phenotypes. *PEAR1* methylation Factor2, characterized by the previously identified megakaryocyte-specific CpG sites, was inversely associated with platelet-monocyte conjugates, P-selectin, and WBC counts, while positively associated with the platelet distribution width (PDW) and with leukocyte CD11b and L-selectin. Moreover, *PEAR1* Factor2 methylation was negatively associated with INFLAscore, a low-grade inflammation score. The latter was partially mediated by the *PEAR1* methylation effect on platelet variables. *PEAR1* methylation association with WBC measurements and INFLAscore was confirmed in the independent cohort FLEMENGHO.

**Conclusions:**

We report a significant link between epigenetic signatures in a platelet functional gene and inflammation-dependent platelet function variability measured in two independent cohorts.

## Introduction

Platelet-endothelial aggregation receptor 1 (PEAR-1) is a membrane receptor involved in cell-cell interactions, particularly expressed in platelets, megakaryocytes, and endothelial cells. PEAR-1 sustains activation of the platelet integrin α_IIb_β_3_ through its src family kinase (c-Src)-dependent phosphorylation that stabilizes platelet aggregate formation [1]. The direct activation of PEAR-1 not only by its pentameric ligand, the FcεR1α chain, but also by anti-PEAR-1 antibodies, dextran sulfate, synthetic glycopolymers, and natural fucoidans triggers potent platelet aggregation [1–4]. Numerous large studies have identified *PEAR1* genetic variants as determinants of platelet response/function variability, both in the general population and in cohorts with cardiovascular outcomes [5–29], suggesting that PEAR-1 may be a signaling component, capable of modulating several functional platelet pathways in physiological conditions, but also in the context of anti-platelet therapy and cardiovascular disease. This seems to be the case in particular for rs12041331 and rs12566888, 2 *PEAR1* variants in linkage disequilibrium (LD) located in intron 1 of the *PEAR1* gene locus [30]. In particular, *PEAR1* rs12041331 G/A substitution leads to lower platelet PEAR1 expression [6] and reduces endothelial cell migration in carriers of the A allele [31], while a negative association of rs12566888 with WBC, neutrophil, and monocyte numbers in a large-scale Exomechip analysis has been reported by Eicher and colleagues [25]. The latter opened up the possibility for a pleiotropic role of *PEAR1* in influencing not only platelet function variability but also hematopoiesis at large. Indeed, *PEAR1* expression increases during megakaryocyte (MK) differentiation and *PEAR1* knock-down CD34^+^ cells show higher proliferation of immature MKs, whereas terminal MK maturation (proplatelet formation) is not affected in the absence of PEAR-1 [32]. In addition, expression profiling on normal human bone marrow sections also showed transient PEAR1 positivity in myeloid precursors, yet absent in mature granulocytes [32].

We have previously identified a region within the first untranslated exon of the *PEAR1* gene that, towards the later stages of MK specification, undergoes a significant increase of DNA methylation level in parallel to *PEAR1* expression [30]. We found the same region to be differentially methylated between megakaryocyte and endothelial cells and to be part of a *superenhancer* that coordinates expression of multiple genes involved in cell cycle and cell proliferation through long-range chromosome interactions [33]. This type of epigenetic regulation contributes to the fine-tuning of *PEAR1* expression, but it remains unclear, at the population level, whether *PEAR1* epigenetic variability would contribute to explain variability of platelet function and would also have an impact on hematopoiesis and leukocyte function.

In this study, we investigated *PEAR1* methylation as a marker of platelet and leukocyte formation, their activation and cross-talk, using DNA samples from a family-based cohort study (the Moli-family study) [34–36], characterized by a large set of hematological activation markers. Our major results were replicated in a second independent population-based cohort (the FLEMENGHO study) [37–39].

## Results

Demographics of the population studied are shown in Table 1. Blood cell counts, platelet, and leukocyte activation markers are reported in Table 2.

**Table 1:** General characteristics of Moli-family participants

Characteristic	Moli-family
Number (%) of participants	605
Women	330 (54.5)
Current smoking	165 (27.4)
Drinking alcohol^a^	122 (21.8)
Hypertension	206 (34.2)
Diabetes	36 (6.0)
Mean (± SD) characteristic	
Age, years	42.3 ± 18.8
Body mass index, kg/m^2^	26.5 ± 5.4
Blood pressure, mm Hg	
Systolic	130.2 ± 20.6
Diastolic	77.1 ± 11.6
Total serum cholesterol, mmol/L	5.06 ± 1.10
HDL cholesterol, mmol/L	1.49 ± 0.38
Total-to-HDL cholesterol ratio	3.56 ± 1.03

HDL indicates high-density lipoprotein. Hypertension was a blood pressure of ≥ 140 mmHg systolic or ≥ 90 mmHg diastolic or use of antihypertensive drugs. Diabetes mellitus was a fasting plasma glucose level of ≥ 7.0 mmol/L or use of antidiabetic agents

^a^Defined as drinking at least 15 g of alcohol daily

**Table 2:** Platelet and WBC variable distribution in the Moli-family cohort

Variable	Moli-family (*n* = 605)
Platelet measurements	
Platelet count, 10^9^/L	249.4 ± 60,6
Mean platelet volume, fL	8.59 ± 0.98
Plateletcrit, %	0.21 ± 0.05
Platelet distribution width, %	16.4 ± 0.6
Soluble P-selectin, μg/L	82.7 ± 38.0
Platelet P-selectin, %	2.76 ± 3.65
Platelet/monocytes aggregates, %	7.24 ± 8.62
Platelet/PMN aggregates, %	4.32 ± 5.02
Inflammation measurements	
White blood cell count, 10^9^/L	6.19 ± 1.52
Neutrophils, %	61.0 ± 8.0
Lymphocyte, %	32.0 ± 7.0
Neutrophil/lymphocyte ratio	2.08 ± 0.83
Monocyte, %	7.0 ± 2.0
Red blood cell, 10^12^/L	4.89 ± 0.5
Platelet/lymphocyte ratio	135.9 ± 47.6
INFLAscore*	− 0.23 ± 5.59
C-reactive protein, mg/mL	1.7 ± 1.74
Monocyte L-selectin, %	10.4 ± 7.2
PMN L-selectin, %	25.7 ± 21.3
Monocyte CD11b, %	51.5 ± 24.1
PMN CD11b, %	44.0 ± 25.1

*INFLAscore is calculated as follows: 10 tiles of each biomarker levels (CRP, WBC, platelets, G/L ratio) were generated. For all four components, being in the highest deciles (7 to 10) gave a score which increased from 1 to 4, while being in the lowest deciles (1 to 4) was negatively scored from − 4 to − 1. Being in the deciles 5 or 6 got zero points. In such a way, the INFLA-score ranges from − 16 to 16 and comes up as the sum of the four biomarkers. An increase in the score represented an increase in low-grade inflammation intensity

**Fig. 1:**
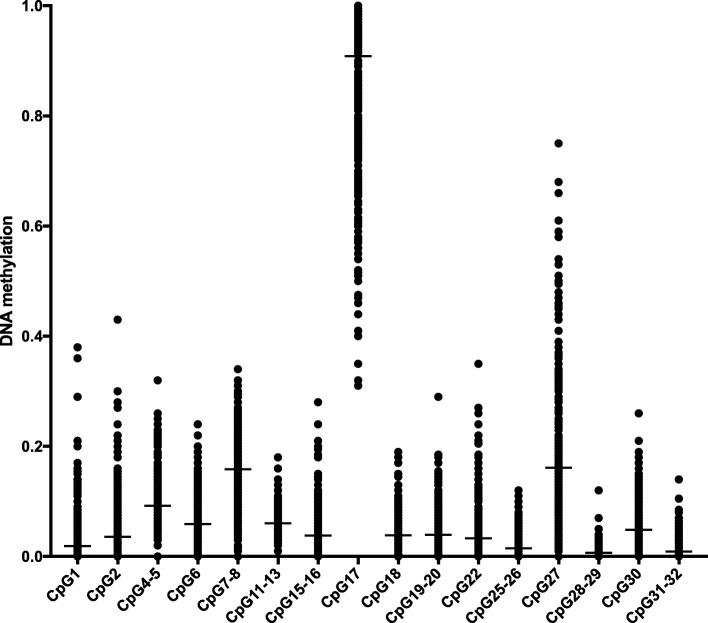
PEAR1 CGI1 DNA methylation distribution in the Moli-family cohort. Dot-plot representation of PEAR1 CpG unit (depicted on the *Y* axis) methylation distribution across the Moli-family participants (*N* = 605). Mean is shown for each unit as a black bar

After removing duplicate units and units with standard deviation (SD) between replicates higher than 5% as described [30, 40, 41], we obtained a total of *PEAR1* 16 CpG units for further analysis. These *PEAR1* CpG sites, identified for the Moli-family cohort, include the previously identified megakaryocyte-specific CpG sites [30]. The exact genomic location of each CpG site in *PEAR1* is reported in the Additional file 1: Table S1. For each of these sites, Fig. 1 shows the distribution of the fractional degree of methylation, uncovering a wide variation in methylation within and between different sites.

After exclusion of failing samples, a total of 605 Moli-family participants were included for further analyses. Mean and SD of *PEAR1* CpG sites methylation ranged from 0.6 ± 1.0% for CpG28–29 up to 90.8 ± 14.1% for CpG17. Only CpG7–8, 17, and 27 had methylation averages across the population higher than 10%, while all the other 13 CpG units appeared less methylated (0 to 10%) (Fig. 1 and Additional file 1: Table S1). Several of the examined CpGs showed strong inter-correlations (Additional file 1: Table S2).

Because *PEAR1* CpG site methylation showed significant inter-correlations, we used principal component analysis (PCA) with the aim of identifying common underlying factors (patterns) that explain the largest variation in methylation. For this analysis, 370 individuals were included having all the *PEAR1* CpG units successfully measured. Three-, four-, and five-factor solutions were discarded on the basis of the scree plot. Two main methylation factors finally emerged with PCA. Factor1 is characterized by high positive loadings of CpGs 4–5, 6, 11–13, 17, 18, 27, and 30, and Factor2 by that of CpGs 7–8, 15–16, 19–20, 22, 25–26, and 31–32 (Additional file 1: Table S3). Three more CpG units (CpG1, CpG2, and CpG28–29) did not appear to contribute to any of the identified methylation factors; therefore, they were included in further analyses as separate methylation variables (Table 4 and Additional file 1: Table S4).

### PEAR1 methylation is associated with ex vivo platelet-monocyte mixed aggregates

Linear mixed regression analysis was performed to assess the relation between *PEAR1* methylation patterns (Factor1, Factor2, CpG1, CpG2 and CpG28–29) and platelet activation phenotypes, considering whole blood baseline platelet P-selectin and platelet-monocyte or platelet-leukocyte mixed conjugates already measured in the cohort [34]. We found that platelet-monocyte, but not platelet-polymorphonuclear cell (PMN), mixed conjugates were consistently negatively associated with *PEAR1* Factor2 methylation (Additional file 1: Table S4) with the association explaining 3.5% of platelet-monocyte aggregate variability (Additional file 1: Table S4). The association between *PEAR1* Factor2 methylation and platelet P-selectin almost reached significance with a *p* value of 0.08. *PEAR1* Factor1, CpG1, CpG2, and CpG28–29 methylation was not associated with any of the platelet-specific variables measured (Additional file 1: Table S4).

In a previous study, focused on the methylation of the same *PEAR1* region, we identified some specific CpG sites that significantly changed their methylation status according to *PEAR1* expression during differentiation of megakaryocyte precursors (CpG4–5, 7–8, 11–13, 15–16, 19–20, 22) [30]. Based on that finding, and because *PEAR1* Factor2 mainly depends on these MK-specific CpG sites (Additional file 1: Table S3), we also performed a specific CpG methylation analysis considering the 16 *PEAR1* CpG units as single measures in the Moli-family cohort. By using this type of approach, we identified CpG sites 7–8 and 19–20 as the most significantly associated *PEAR1* sites with platelet P-selectin and platelet-PMN aggregates (Additional file 1: Table S5).

### PEAR1 methylation is associated with white blood cell measurements

We investigated the possible association of *PEAR1* methylation with blood cell counts in the Moli-family cohort. While no significant association was found with platelet count, mean platelet volume (MPV), or plateletcrit (Pct), *PEAR1* Factor2 methylation was positively associated with platelet distribution width (PDW) (Table 3), a measure of platelet heterogeneity and indirect estimate of megakaryopoiesis variation. This association explained 2.6% of PDW variability (Table 3).

**Table 3:** PEAR1 methylation is significantly associated with PDW, WBC, lymphocyte, monocyte, and neutrophil counts in the Moli-family cohort

	Factor1				Factor2				P1				P2				P28_29			
	Beta	SE	*p* value	*R*-square	Beta	SE	*p* value	*R*-square	Beta	SE	*p* value	*R*-square	Beta	SE	*p* value	*R*-square	Beta	SE	*p* value	*R*-square
Platelets	− 0.088	0.048	0.071	0.006	0.083	0.052	0.109	0.007	0.009	0.038	0.800	0.000	− 0.011	0.042	0.788	0.000	− 0.025	0.038	0.500	0.001
MPV	− 0.033	0.053	0.530	0.001	0.001	0.058	0.993	0.000	0.017	0.040	0.679	0.000	− 0.004	0.044	0.928	0.000	0.048	0.041	0.233	0.004
Pct	− 0.105	0.049	0.034	0.008	0.100	0.053	0.058	0.007	0.014	0.038	0.790	0.000	− 0.001	0.042	0.979	0.000	− 0.005	0.038	0.888	0.000
PDW	0.023	0.056	0.677	0.002	0.145	0.058	*0.013*	0.026	0.052	0.043	0.223	0.002	− 0.052	0.045	0.249	0.000	− 0.010	0.041	0.800	0.000
WBC	− 0.047	0.056	0.397	0.001	− 0.123	0.058	*0.036*	0.020	− 0.018	0.041	0.657	0.000	− 0.066	0.045	0.143	0.008	0.001	0.041	0.989	0.000
Lymphocytes	0.126	0.053	*0.019*	0.002	0.210	0.055	*0.0002*	0.009	0.027	0.041	0.521	0.000	0.097	0.044	*0.028*	0.001	0.006	0.041	0.887	0.000
Monocytes	− 0.085	0.052	0.105	0.005	0.239	0.055	*< 0.0001*	0.023	0.075	0.037	*0.045*	0.005	0.040	0.041	0.327	0.001	0;015	0.037	0.691	0.000
Neutrophils	− 0.105	0.055	0.081	0.003	− 0.275	0.055	*< 0.0001*	0.046	− 0.049	0.041	0.228	0.001	− 0.104	0.044	*0.019*	0.009	− 0.008	0.040	0.851	0.000

Model adjusted by age, gender, BMI, smoking (being an ever smoker and pack-years), drinking (a drinker was defined as drinking daily more than 15 g of alcohol), being a proband for family recruitment, being a member of MI family as fixed effects, and family as a random effect. *R*-squares were calculated using a linear regression model including covariates used as fixed effect in the mixed models. Standardized values of phenotypes and methylation are reported. Significant *p* values are reported in italics

In addition to that, we identified an inverse relationship between both *PEAR1* Factor2 and total white blood cell (WBC) counts and neutrophil percentage and a positive one with both lymphocyte and monocyte percentages (Table 3). *PEAR1* methylation could explain 4.6% of neutrophil percentage variability (Table 3).

When adjusting the analysis for platelet variables (platelet count, PDW, platelet P-selectin, platelet/monocyte, and platelet/PMN mixed aggregates), all WBC measurements still remained significantly associated with Factor2 with increased significance and effect of the associations (Table 4 Model B). When removing the platelet influence on this association, *PEAR1* Factor2 methylation could explain 5.1, 6.9, and 7.9% of lymphocyte, monocyte, and granulocyte variability, respectively (Table 4 Model B), suggesting that the association between *PEAR1* methylation and these cells was independent of thrombopoiesis or platelet activity.

**Table 4:** Platelets do not influence the association between Factor2 *PEAR1* methylation and WBC counts in the Moli-family cohort

	Model A				Model B			
	Beta	SE	*p* value	*R*-square	Beta	SE	*p* value	*R*-square
WBC	− 0.123	0.058	*0.036*	0.020	− 0.150	0.054	*0.006*	0.025
Lymphocytes	0.210	0.055	*0.0002*	0.009	0.224	0.056	*< 0.0001*	0.051
Monocytes	0.239	0.055	*< 0.0001*	0.023	0.268	0.057	*< 0.0001*	0.069
Granulocytes	− 0.275	0.055	*< 0.0001*	0.046	− 0.286	0.056	*< 0.0001*	0.079

Model A adjusted by age, gender, BMI, smoking (being an ever smoker and pack-years), drinking (a drinker was defined as drinking daily more than 15 g of alcohol), being a proband for family recruitment, being a member of MI family as fixed effects, and family as a random effect. Model B is Model A +adjustment by platelets, PDW, platelet P-selectin, platelet/monocyte, and platelet/PMN. *R*-squares were calculated using a linear regression model including covariates used as fixed effect in the mixed models. Standardized values of phenotypes and methylation are reported. Significant *p* values are reported in italics

### PEAR1 methylation is associated with markers of inflammation

Because *PEAR1* methylation appeared to be involved at a different level in platelet and WBC variability, and because both platelets and leukocytes are mediators of cellular inflammation, we have further investigated whether PEAR1 methylation would be linked to inflammation markers, available in the Moli-family cohort. For this purpose, we studied the association between *PEAR1* methylation and markers of platelet and leukocyte activation and C-reactive protein (CRP) levels.

*PEAR1* Factor2 methylation was strongly and significantly associated with soluble P-selectin levels following an inverse relationship (Table 5 Model A). Interestingly, this association could explain 10% of plasma P-selectin variability (Table 5 Model A). Soluble P-selectin not only results from platelet activation, but can also be of endothelial cell origin; therefore, we corrected this association for platelet-specific covariates (Table 5, Model B). We observed that, while almost no change occurred considering the effect (ß values) of *PEAR1* methylation on plasma P-selectin levels, the variability of the same marker explained by the methylation dropped from 10 to 5% (Table 5, Model B).

**Table 5:** *PEAR1* Factor2 methylation association with markers of cell and plasmatic inflammation partially depends upon platelet activation n the Moli-family cohort

	Model A				Model B			
	Beta	SE	*p* value	*R*-square	Beta	SE	*p* value	*R*-square
Soluble P-selectin	− 0.197	0.057	*< 0.0001*	0.100	− 0.207	0.054	*0.0002*	0.051
Monocyte CD11b	0.034	0.057	0.549	0.005	0.021	0.057	0.719	0.0004
PMN CD11b	0.122	0.058	*0.035*	0.043	0.144	0.058	*0.014*	0.020
Monocyte L-selectin	0.155	0.065	*0.019*	0.021	0.127	0.066	0.056	0.013
PMN L-selectin	0.151	0.061	*0.015*	0.059	0.183	0.063	*0.004*	0.030
CRP	− 0.078	0.053	0.145	0.016	− 0.087	0.054	0.112	0.009
INFLAscore^a^	− 0.143	0.058	*0.014*	0.026	− 0.111	0.059	0.057	0.012

Model A adjusted by age, gender, BMI, smoking (being an ever smoker and pack-years), drinking (a drinker was defined as drinking daily more than 15 g of alcohol), being a proband for family recruitment, being a member of MI family as fixed effects, and family as a random effect. Model B is Model A +adjustment by platelets, PDW, platelet P-selectin, platelet/monocyte, and platelet/PMN. ^a^INFLAscore was adjusted only for PDW, soluble P-selectin, platelet P-selectin, platelet-monocyte aggregates, and platelet-PMN aggregates, but not platelet number, as this variable is accounted in its formula. *R*-squares were calculated using a linear regression model including covariates used as fixed effect in the mixed models. Standardized values of phenotypes and methylation are reported. Significant *p* values are reported in italics

*PEAR1* Factor2 methylation had a significant positive relation with expression of CD11b on PMNs and of L-selectin on both monocytes and PMNs (Table 5). However, when the model was adjusted for platelet-dependent covariates (Table 5 Model B), these associations partially decreased in significance, their effect, and the percentage of variability, despite residual associations with *PEAR1* methylation, indicative of a relation between *PEAR1* methylation and platelet-dependent but also platelet-independent inflammation.

We did not find any association between *PEAR1* Factor2 and CRP level (Table 5, Model A), but we observed an inverse association with INFLAscore, a validated composite marker of low grade inflammation status [42, 43] calculated from the combination of plasmatic and cellular biomarkers (CRP levels, platelet and total WBC counts, and granulocyte/lymphocyte (G/L) ratio, Table 1). This association that explained the 2.6% of total INFLAscore variability in the Moli-family cohort, also disappeared when the model was adjusted for platelet function variables (PDW, soluble P-selectin, platelet P-selectin, platelet/monocyte and platelet/PMN) (Table 5, Model B). On the contrary, the association of *PEAR1* Factor2 methylation with platelet-monocyte mixed aggregates was still significant and of similar effect when adjusting the multivariate model for CRP (Additional file 1: Table S6, Model B).

### Replication study in the FLEMENGHO cohort

To replicate the Moli-family findings, we have studied *PEAR1* methylation in 1002 participants belonging to the FLEMENGHO cohort [37–39]. General characteristics and blood cell count variables of FLEMENGHO participants are reported in Additional file 1: Table S7. Because *PEAR1* methylation distribution slightly differs among the two studies, possibly due to underlying population differences, PCA analysis gave rise to different factors in FLEMENGHO compared to Moli-family. Therefore, we have used single CpG analysis to replicate the Moli-family findings. Interestingly, methylation at CpG4–5, CpG7–8, CpG11–13, and CpG 19–20 in the FLEMENGHO cohort was significantly associated with the percentage of neutrophils and lymphocytes in the same fashion as in the Moli-family cohort (Additional file 1: Table S8). Measurements of L-selectin expression on monocytes and PMN were not available for FLEMENGHO participants; however, circulating L-selectin was measured [44, 45]. Using the same model, circulating L-selectin levels in FLEMENGHO were consistently and inversely associated with methylation at CpG7–8 and CpG19–20 in accordance with Moli-family data (Additional file 1: Table S8). In addition, INFLAscore was also negatively associated with methylation estimates at CpG7.8 and CpG11.13 in FLEMENGHO, confirming Moli-family results.

## Discussion

DNA methylation estimates at the *PEAR1* CpG island (CGI) 1 region are associated with variability of a series of platelet and WBC parameters ranging from function/activation (P-selectin, L-selectin, CD11b) to cell heterogeneity (PDW) and number (WBC, monocytes, and granulocytes).

*PEAR1* was first identified as a genetic determinant of platelet function variability mostly in in vitro assays and after platelet stimulation [5–12, 14–16, 25–27, 29]. PEAR-1 in platelets supports activation of the α_IIb_β_3_ integrin [1] and influences MK precursor proliferation through control of the PI3K-PTEN pathway [32]. We have already shown that *PEAR1* methylation controls *PEAR1* expression during megakaryopoiesis [30, 46] and is lower in carriers of rs12041331, a CpG-SNP whose presence alters the methylation pattern of the *PEAR1* intron1 region causing differential binding of nuclear proteins [30].

We report now, for the first time, that DNA methylation at the same locus is associated with platelet activation variability, at baseline in a well-defined population. We observed an inverse relation between *PEAR1* methylation and platelet-monocyte mixed cell conjugates and with platelet P-selectin at baseline conditions (Additional file 1: Tables S4 and S5). Interestingly, we found the CpG sites mostly involved in this association to be among the ones described in the MK-specific experiments [30]. In line with our findings, Wurtz and colleagues [14] reported rs12041331 (associated with lower methylation) to be linked to lower platelet aggregation but increased P-selectin expression. Circulating monocyte-platelet aggregates have been demonstrated to be a sensitive marker of in vivo platelet activation (together with platelet surface P-selectin) [47]. Platelet-leukocyte conjugates measured in the Moli-family cohort, as used in the present analysis, are mainly formed upon platelet but not leukocyte activation [34]. In addition, *PEAR1* methylation-dependent platelet activation (platelet-monocyte aggregates) is only very mildly affected by CRP levels (Additional file 1: Table S6), while yet having a role in influencing inflammation (Table 5). Indeed, platelet-leukocyte conjugates have served as a specific marker of platelet activation/function in several thrombo-inflammatory conditions [48–54].

We did not find any association between PEAR1 (methylation) and platelet counts; however, we did observe a significant positive relation with PDW, a measure of platelet size heterogeneity, with possible consequences on platelet formation and function [55, 56]. In our study, we could not clarify whether PEAR-1 plays a role in controlling the PDW, because this is not demonstrated by our analysis; however, we have previously observed that individuals carrying the A-allele of rs12041331, therefore showing lower methylation levels at CGI1, also had a lower number of big MK colony forming units (CFU) compared to GG homozygotes [30].

Very recently, a possible role of PEAR-1 in controlling hematopoiesis at large has been suggested by the study of Eicher and colleagues [25]. In this meta-analysis, rs12566888, a *PEAR1* variant in LD with rs12041331, was significantly associated with a reduced number of total WBC, monocytes, and neutrophils. PEAR-1 (JEDI) was already described to have a role in the fine regulation of the early stages of hematopoietic differentiation, presumably through the Notch pathway, beside its role as phagocytic receptor involved in clearing dead sensory neurons [57]. Our earlier evidence demonstrated transient *PEAR1* expression in myeloid precursors [32]. Further supporting these data, we have recently shown that the *PEAR1* CGI1 region is part of a larger enhancer that in hematopoietic precursors mediates interactions with genes that are very active in cell differentiation because they regulate protein synthesis, cell cycle, and cell proliferation, possibly via DNA methylation changes [33]. All these data point to a possible role of PEAR-1 in driving variability of formation of hematopoietic cells and are in line with the association of *PEAR1* methylation with white blood cell counts in both Moli-family and FLEMENGHO cohorts, a link that seems to be independent from platelets.

In accordance with the identified link between *PEAR1* methylation, platelet, and WBC measurements, Factor2 methylation was also associated with other inflammatory markers. *PEAR1* Factor2 methylation can explain about 10% of soluble P-selectin variability, which is remarkable for a platelet surface molecule, considered to have a modulating role in platelet activation. Yet, the P-selectin association may also reflect *PEAR1* methylation-mediated effects in vascular endothelial cells that abundantly express both PEAR1 and P-selectin [1, 58, 59]. Indeed, when correcting the association for platelet-specific variables, the variability of plasma P-selectin levels explained by PEAR1 methylation was half-sized, suggesting a possible alternative (endothelial) cell origin of plasma P-selectin. In accordance with the latter, the rs12041331 variant was also described as a determinant of endothelial cell function [31] and DNA methylation at *PEAR1* is associated with differential *PEAR1* expression in different endothelial cell types [33]. We also found in the Moli-family cohort a significant association with the leukocyte specific activation markers CD11b and L-selectin and INFLAscore, a composite score integrating both cell and plasmatic inflammation parameters (Table 5). Adjustment for platelet variables, however, partially affected these associations, suggesting that platelet activation determined by *PEAR1* methylation variability can have an impact on inflammation, in agreement with the role of platelets as an inflammatory mediator [60, 61]. Together with P- and E-selectin, L-selectin mediates the first adhesive step during inflammation [59, 62] and is rapidly shed from the cell surface upon leukocyte activation in order to promote cell migration after extravasation [63]. This is in line with the negative association of PEAR1 methylation with circulating L-selectin levels measured in the replication cohort FLEMENGHO. Together, this evidence suggests that the observed association could in part result from platelet-dependent P-selectin-PSGL-1 interactions on leukocytes, subsequently enhancing leukocyte activation and L-selectin shedding [64, 65].

In conclusion, we report for the first time that *PEAR1* methylation is a marker of platelet activation and WBC count variability, in turn regulating the underlying inflammatory status of an individual. By analyzing two independent cohorts, we were able to provide the first link between epigenetic gene regulation and platelet biology, and function variability and hitherto platelet-dependent inflammatory processes. Future studies should investigate the role of PEAR-1 in hematopoietic specification, proliferation, and cell function, in relation to its importance for immunity and inflammation regulation and possible thrombo-inflammatory clinical outcomes.

## Materials and methods

### Study population

The Moli-family cohort [35, 36] includes 754 white subjects (≥ 15 years old) from 54 extended pedigrees (23 families with personal or familial history of early myocardial infarction (MI)—MI family—and 31 families without) recruited in the Southern Molise region in Italy. All participants were relatives of index subjects enrolled in the population-based Moli-sani study [66, 67]. In all subjects, a complete medical history and information about smoking and alcohol-drinking habits were obtained via a structured questionnaire. Height, body weight, and blood pressure were measured as described [36, 66]. Blood samples were obtained between 07:00 and 09:00 from participants who had fasted overnight and had refrained from smoking for at least 6 h. Enrollment and data collection were performed as previously described [68]. Of the 754 Moli-family participants, 623 had good quality DNA samples to perform the methylation analysis.

The FLEMENGHO cohort includes 3343 White European participants living in a geographically defined area of Northern Belgium [37, 39, 69]. The follow-up cycles between 2005 and 2015 included 1447 participants, among which 786 and 661 participants had one and two examinations, respectively. Among 2108 examinations, 1266 DNA or blood samples of 1118 subjects had a good quality for methylation measurement. For the analysis, we further excluded subjects with any missing information of blood count (*n* = 37), or CRP (*n* = 37), or soluble L-selectin (*n* = 12). Finally, 1002 FLEMENGHO participants were included in the current analysis.

In both cohorts, individuals with overt inflammatory conditions were excluded from the analysis (CRP levels > 10 mg/dl).

### Flow cytometry and biochemical measurements

#### Moli-family

Mixed platelet-leukocyte conjugates and markers of platelet or leukocyte activation were measured in the Moli-family participants as described [34]. Briefly, venous blood was collected in 3.8% trisodium citrate vacutainer tubes and processed between 10 and 20 min after collection. Whole blood was then either immediately fixed using a commercially available fixative (ThromboFix™, Beckman Coulter Inc.) or stimulated in vitro using both platelet and leukocyte agonists, namely ADP and collagen or fMLP, or LTB4, respectively. Details of the protocol were reported before [34]. Platelet-leukocyte conjugates, platelet P-selectin, leukocyte CD11b, and L-selectin expression were measured in whole blood as described [34]. Platelets, including platelet aggregates, were defined by morphological characteristics and by CD42b positivity. PMN and monocyte populations were defined on the basis of the side scatter (SS) characteristics within the CD45^+^ population [34].

Biochemical analyses were performed in the centralized Moli-sani laboratory. All hematological cytometric analyses were performed by the same cell counter (Coulter HMX, Beckman Coulter, IL Milan, Italy), within 1 h from venipuncture. Soluble P-selectin was measured in stored plasma via the Human P-selectin Platinum Enzyme-linked Immunosorbent Assay (ELISA) kit (Affimetrix, eBioscience). High-sensitivity (hs) CRP was measured in serum as described [43, 67].

#### FLEMENGHO

Hematological cytometric analyses were performed by the Centrum voor Medische Analysen (Herentals, Belgium). Randox Laboratories Ltd. (County Antrim, Northern Ireland, UK) blindly measured CRP and soluble L-selectin using Biochip Array Technology according to the manufacturer’s instructions (Adhesion Molecule and Cerebral II arrays) and a sandwich assay format [44, 45].

### INFLAscore in Moli-family and FLEMENGHO

INFLAscore, which had been previously used within the Moli-sani cohort [42, 43] to evaluate the possible synergistic effect of both plasma and cellular biomarker of inflammation, was calculated in both Moli-family and FLEMENGHO as described: 10 tiles of each biomarker levels (CRP, WBC, platelets, granulocyte/lymphocyte (G/L) ratio) were generated. For all four components, being in the highest deciles (7 to 10) gave a score which increased from 1 to 4, while being in the lowest deciles (1 to 4) was negatively scored from − 4 to − 1. Being in the deciles 5 or 6 got zero points. In such a way, the INFLAscore ranges from − 16 to 16 and comes up as the sum of the four biomarkers. An increase in the score represented an increase in low-grade inflammation intensity.

### DNA methylation analysis

*PEAR1* (CGI1) methylation was evaluated using the Sequenom EpiTYPER MassARRAY (Agena) platform as described [30, 40, 41] on white blood cell DNA from 605 Moli-family and 1002 FLEMENGHO participants.

Bisulfite treatment was conducted on 1 μg of genomic DNA using the MethylDetector kit (Active Motif) according to the manufacturer’s instructions, except for the incubation protocol during the conversion, performed for a total of 16 h as described [70]. The amplicon to study *PEAR1* methylation was designed using the Sequenom EpiDesigner software (http://www.epidesigner.com/) [30]. All PCR amplifications were performed in duplicate. For the CpG-specific analysis, data were discarded when the duplicate measurements had a SD equal to or greater than 5% [30, 40, 41]. Sequenom peaks with reference intensity above 2 and overlapping units were excluded from the analysis [30, 40, 41]. To exclude possible intra-plate differences, a sample of K562 DNA, with known *PEAR1* methylation profile (around 90%), was carried on in each plate.

### Statistical analysis

All analyses were performed using SAS/STAT software (Version 9.4 for Windows©2009. SAS Institute Inc. and SAS are registered trademarks of SAS Institute Inc., Cary, NC, USA).

We used principal component analysis (PCA) on the correlation matrix of the *PEAR1* 16 CpG sites to identify *PEAR1* methylation patterns in the Moli-family cohort [71]. We characterized the factors using the *PEAR1* methylation sites with an absolute factor loading greater than 0.25. Criteria for number of factor selection were eigenvalue > 1.0 as revealed by the scree test. Each subject received a factor score, calculated by summing the observed methylation site values, each weighted by factor loadings. Linear mixed regression models were used to evaluate associations between *PEAR1* methylation factors or single CpG sites and phenotypes. Age, gender, BMI, smoking (being an ever smoker and pack-years), alcohol drinking (a drinker was defined as drinking daily more than 15 g of alcohol), being a proband for family recruitment, and being a member of MI family were treated as fixed effects, and family stratification as a random effect. *R*-squares were calculated using a linear regression model including covariates used as fixed effect in the mixed models. Platelet parameters or CRP were used where appropriate as indicated below to verify their potential role as confounders on the effect of *PEAR1* methylation on white blood cell or platelet parameters.

To replicate the Moli-family findings in FLEMENGHO, we used a multivariate regression model with age, gender, BMI, smoking (being an ever smoker and pack-years), and drinking (a drinker was defined as drinking daily more than 15 g of alcohol) as covariates.

A false discovery rate (FDR) method (Benjamini-Hochberg) were used to adjust *p* values for multiple testing with a *p* value (pFDR) < 0.05 considered as statistically significant.

## Supplementary information

**Additional file 1.** Tables S1-S8.

## Data Availability

All data analyzed in this study are included in this published article. Raw data used for the analysis are availbale upon request.

## Abbreviations

CFU: Colony forming units
CGI: CpG island
CRP: C-reactive protein
c-Src: src family kinase
FDR: False discovery rate
G/L: Granulocyte/lymphocyte
Hs: High sensitivity
LD: Linkage disequilibrium
MI: Myocardial infarction
MK: Megakaryocyte
MPV: Mean platelet volume
PCA: Principal component analysis
Pct: Plateletcrit
PDW: Platelet distribution width
PEAR-1: Platelet-endothelial aggregation receptor 1
PMN: Polymoprphonuclear cell
SD: Standard deviation
WBC: White blood cell
